# The primordial germ line is refractory to perturbations of actomyosin regulator function in *C. elegans* L1 larvae

**DOI:** 10.17912/micropub.biology.000432

**Published:** 2021-08-04

**Authors:** Jack Bauer, Léa Lacroix, Jean-Claude Labbé

**Affiliations:** 1 Institute for Research in Immunology and Cancer (IRIC), Université de Montréal, C.P. 6128, Succ. Centre-ville, Montréal, QC, H3C 3J7, Canada; 2 Department of Pathology and Cell Biology, Université de Montréal, C.P. 6128, Succ. Centre-ville, Montréal, QC, H3C 3J7, Canada

## Abstract

Cytokinesis, the separation of daughter cells at the end of mitosis, relies on the coordinated activity of several regulators of actomyosin assembly and contractility (Green et al. 2012). These include the small GTPase RhoA (RHO-1) and its guanine-nucleotide exchange factor Ect2 (ECT-2), the scaffold protein Anillin (ANI-1), the non-muscle myosin II (NMY-2), the formin CYK-1 and the centralspindlin complex components ZEN-4 and CYK-4. These regulators were also shown to be required for maintenance of *C. elegans* germline syncytial organization by stabilizing intercellular bridges in embryos and adults (Amini et al. 2014; Goupil et al. 2017; Green et al. 2011; Priti et al. 2018; Zhou et al. 2013). We recently demonstrated that many of these regulators are enriched at intercellular bridges in the small rachis (proto-rachis) of L1-stage larvae (Bauer et al. 2021). We sought to assess whether these contractility regulators are functionally required for stability of intercellular bridges and maintenance of the primordial germ line syncytial architecture in L1-stage *C. elegans* animals. Here we report that temperature-sensitive alleles, RNAi-mediated depletion and latrunculin A treatment are largely ineffective to perturb actomyosin function in the L1-stage primordial germ line.

**Figure 1. Perturbation of actomyosin function in the C. elegans primordial germ line:**
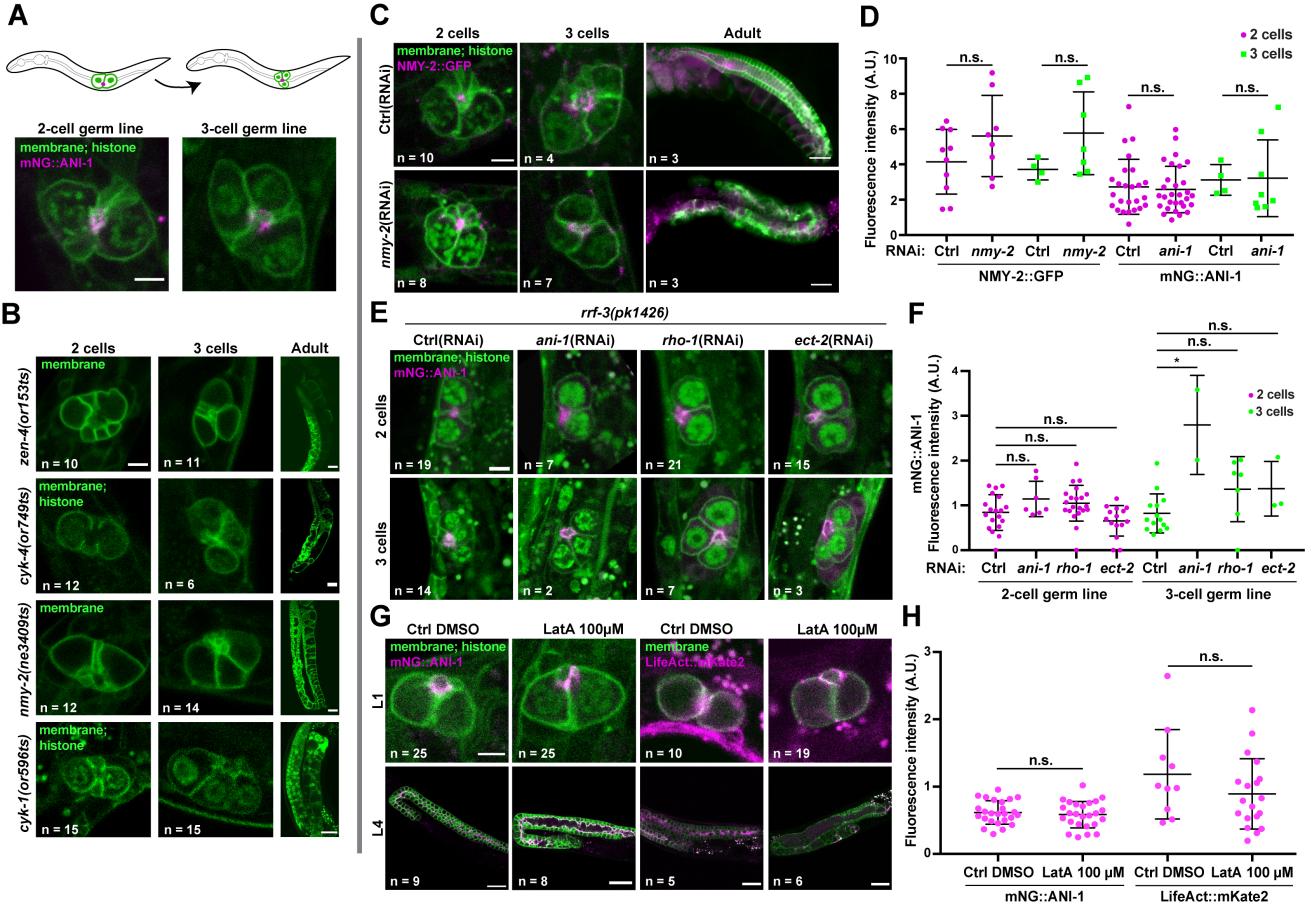
Schematic representation (top) and confocal images (bottom) of the 2-cell (left) and 3-cell (right) primordial germ line in control L1-stage animals. **B.** Confocal images of the primordial germ line containing 2 (left) or 3 (middle) germ cells in L1-stage animals bearing temperature-sensitive alleles for *cyk-4(or749ts)*, *zen-4(or153ts),* *nmy-2(ne3409ts)* or *cyk-1(or596ts)* that were upshifted at 26°C for 12h. The panels on the right show the adult germ line after animals of each genotype were grown at 26°C. **C.** Images of the 2-cell (left), 3-cell (middle) and adult (right) germ line in animals co-expressing NMY-2::GFP (magenta) and markers for membranes and histones (green) that were soaked at the L1 stage in control (top) or *nmy-2* (bottom) dsRNA. **D.** Measured fluorescence intensity of NMY-2::GFP and mNG::ANI-1 at the proto-rachis of L1-stage animals soaked respectively in control, *nmy-2* or *ani-1* dsRNA (RNAi). **E.** Confocal images of the 2-cell (top) and 3-cell (bottom) germ line in *rrf-3(pk1426)* mutant animals co-expressing mNG::ANI-1 (magenta) and markers for membranes and histones (green) that were soaked at the L1 stage in control (far left), *ani-1* (middle left), *rho-1* (middle right) or *ect-2* (far right) dsRNA. **F.** Measured fluorescence intensity of mNG::ANI-1 at the proto-rachis of L1-stage animals soaked respectively in control, *ani-1*, *rho-1*,or *ect-2* dsRNA (RNAi). **G.** Confocal images of the germ line in L1 (top) and L4 (bottom) animals co-expressing mNG::ANI-1 (left) or LifeAct::mKate2 (right; magenta) and markers for membranes and histones (green) after treatment with 100 µM latrunculin A or solvent alone (control DMSO). **H.** Measured fluorescence intensity of mNG::ANI-1 and LifeAct::mKate2 at the proto-rachis of L1-stage animals treated with 100 µM of latrunculin A (LatA) or solvent alone (DMSO control). In all panels, images shown are sum projections of 3 confocal slices, membranes are marked with TagRFP-, GFP- or mNG-tagged PH^PLCδ^ and histones are marked with mCh-HIS-58 (see strain table for details). For all images of L1 animals, scale bar = 3 µm. For all images of L4 and adult animals, scale bar = 30 µm. For all graphs, black lines represent mean ± standard deviation, and statistical analyses were done using a one-way ANOVA test with a Tukey *post hoc* test (n.s. = p > 0.05; * = p < 0.001).

## Description

To perturb actomyosin function in the primordial germ line, we first monitored germ line organization in L1-stage animals bearing temperature-sensitive (ts) alleles in genes encoding actomyosin regulators and that were reported to interfere with cytokinesis during embryogenesis (Davies *et al.* 2014). Previous work demonstrated that the initial stages of germline expansion occur normally in *cyk-4*(ts) and *zen-4*(ts) animals raised at restrictive temperature from the L1 stage (Lee *et al.* 2018). We found that primordial germ line organization in *cyk-1*(ts), *nmy-2*(ts), *cyk-4*(ts) or *zen-4*(ts) L1 larvae maintained at restrictive temperature for 12h was no different than control (**Figure 1A-B**). Furthermore, the first primordial germ cell (PGC) division occurred normally upon feeding these animals at restrictive temperature with typical bacterial food (*E. coli* OP50). As noted previously (Lee *et al.* 2018), germ line disorganization and sterility were observed in all cases when animals reached adulthood (**Figure 1B**).

We then used RNAi to deplete actomyosin regulators in L1 larvae expressing NMY-2::GFP or mNG::ANI-1, as well as fluorescent markers for membrane and histone. We found that soaking L1 animals for 24h in a dsRNA solution against *nmy-2* or *ani-1* did not significantly perturb primordial germ line organization nor decreased fluorescence levels of these proteins compared to control L1-stage larvae (**Figure 1C-D**). Feeding of these soaked animals with OP50 revealed that the first PGC division occurred normally, and germ line disorganization was observed when these animals reached adulthood (**Figure 1C**). This demonstrates that the RISC complex had effectively been engaged by dsRNA treatment at the L1 stage but that the phenotype only manifested itself later in development. Similar results (lack of phenotype in L1 larvae, potent phenotype in adults) were obtained when we soaked RNAi-hypersensitive *rrf-3(pk1426)* mutants in dsRNA against *ani-1*, *rho-1* or *ect-2* for 24h (**Figure 1E-F**).

Finally, we treated L1 larvae expressing mNG::ANI-1 or LifeAct::mKate2 (marking F-actin) with the actin depolymerizing drug latrunculin A and scored primordial germ line organization. We found that incubating L1 larvae for 3-5 hours in a solution of 100 µM latrunculin A did not result in significant primordial germ line disorganization and the fluorescence levels of either marker at the proto-rachis remained unchanged compared to control (**Figure 1G-H**). As shown previously (Priti *et al.* 2018), latrunculin A treatment of L4 larvae (even with a lower dose of 25 µM) resulted in an extensive collapse of the germ cell intercellular bridges (**Figure 1G**), demonstrating that the drug is effective.

Together with previous work (Lee *et al.* 2018), our results demonstrate that perturbing the function of actomyosin contractility regulators in the *C. elegans* primordial germ line is difficult to achieve at the L1 stage by means of ts alleles, RNAi or latrunculin A treatment. The reasons for this are unclear and could vary depending on the treatment, yet we consider it unlikely that these gene products are dispensable for germline development. Notably, RNAi depletion in PGCs was previously achieved for regulators of the spindle assembly checkpoint (Lara-Gonzalez *et al.* 2019), and our finding that RNAi treatment at the L1 stage results in phenotypes later in development indicates that the RNAi machinery can be engaged in L1 animals. One possibility is that actomyosin regulators within the primordial germ line are organized in a very compact and/or stable manner that makes perturbation difficult, a situation perhaps analogous to microtubule organization at the midbody prior to abscission (Hu *et al.* 2012; Salmon *et al.* 1976). While other approaches for gene depletion could be more effective (e.g. degron-based), this phenomenon will require further investigation.

## Methods

***C. elegans* strain maintenance**

Animals were grown on NGM plates seeded with *E. coli* strain OP50 and maintained at 20°C as described (Brenner 1974), with the exception of temperature-sensitive strains and *rrf-3(pk1426)* mutants that were maintained at 15°C. First stage (L1) larvae were obtained by dissolving gravid hermaphrodites in sodium hypochlorite solution (1.2% NaOCl, 250 mM NaOH) and hatching recovered embryos for 24h at room temperature (or at 15°C for ts strains) in M9 buffer (22.04 mM KH_2_PO_4_, 42.27 mM Na_2_HPO_4_, 85.55 mM NaCl, 1 mM MgSO_4_).

**Imaging**

Animals were immobilized in M9 buffer supplemented with 0.2% tetramisole, mounted on an agarose pad (3% for L4s/adults and 5% L1s), and a coverslip was applied and sealed with VaLaP (1:1:1 Vaseline, lanolin, and paraffin). With one exception, images were acquired with a GaAsP detector at 16-bit depth mounted on a Zeiss LSM880 laser-scanning confocal microscope, controlled by ZEN black 2.1 SP3 software, and using a Plan-Apochromat 63x/1.4 oil DIC M27 objective; images of adult animals in Figure 1B were acquired with an HRM camera mounted on a Zeiss AxioImager Z1 microscope and using a Plan-Apochromat 10x/1.4 NA objective. All images were further processed and analyzed using ImageJ software (National Institutes of Health). The fluorescence intensity of contractility regulators was determined by measuring the raw integrated density of the proto-rachis region in sum projections of z-slices comprising the entire primordial germ line. Fluorescence background was measured in the same sum projections, in regions located in the germ cell cytoplasm (when possible, otherwise next to the PGCs) and subtracted from measurements made at the proto-rachis.

**Temperature-sensitive strain upshifts**

Newly hatched and unfed L1 animals were upshifted at 26°C for 12h in M9 buffer, then transferred to NGM plates seeded with *E. coli* OP50 at 26°C, for 2-3h to image 2-cell germ lines and 5-6h to image 3-cell germ lines. For controls, unfed L1 animals were left at 15°C for 12h, then plated on NGM plates seeded with *E. coli* OP50 at 15°C for 4-5h to image 2-cell germ lines and 9-10h to image 3-cell germ lines.

**dsRNA production**

Bacterial clones targeting the genes *nmy-2* (sjj_F20G4.3), *ani-1* (sjj_Y49E10.19), *rho-1* (cenix:169-h12) and *ect-2* (sjj_T19E10.1a) as well at the L4440 empty vector we used as template in PCR reactions and individual inserts flanked by T7 promoters were amplified using T7 promoter-specific primers. PCR products were purified on columns (Qiagen) and used as template for *in vitro* transcription reactions using the T7 Ribomax Express RNAi System (Promega).

**RNA Interference**

First larval stage (L1) animals were soaked for 24h at 15°C in 2-4 µl of buffer (10.9 mM Na_2_HPO_4_, 5.5 mM KH_2_PO_4_, 2.1 mM NaCl, 4.7 mM NH_4_Cl, 6.3 mM spermidine, 0.11% gelatin) supplemented with 8-20 µg of dsRNA targeting *nmy-2*, *ani-1*, *rho-1* or *ect-*2, as described (Green *et al.* 2011). Animals were then washed 3 times with M9 buffer and allowed to recover in M9 buffer for 24h at 15°C. Animals were either imaged immediately or grown at 15°C on NGM plates seeded with *E. coli* OP50 and imaged after first PGC division or after having reached the adult stage.

**Latrunculin A treatments**

L1- or L4-stage animals were individually picked and incubated for 3-5h in M9 buffer supplemented with either 25 µm or 100 µm of latrunculin A (from a 50 mM stock solution in DMSO). For controls, animals were incubated in M9 buffer supplemented with solvent alone (0.5% or 2% DMSO, respectively).

## Reagents

**Table:** 

**Strain**	**Genotype**	**Available from**
JCC146	cyk-1(or596ts) unc-119(ed3)* ItIs38[pAA1; pie-1/GFP::(PLC1delta1); unc-119 (+)] III; ItIs37 [pAA64; pie-1/mCherry::his-58; unc-119 (+)] IV	Canman lab
OD239	*cyk-4(or749ts) unc-119(ed3) ItIs38[pAA1; pie-1/GFP::(PLC1delta1); unc-119 (+)] III; ItIs37 [pAA64; pie-1/mCherry::his-58; unc-119 (+)] IV*	Oegema lab
UM639	*cpSi20[Pmex-5::TAGRFPT::PH::tbb-2 3’UTR + unc-119(+)] II; zuIs45[nmy-2::NMY-2::GFP + unc-119(+)]; ltIs37 [pAA64; pie-1::mCherry::HIS-58; unc-119(+)] IV*	This study
UM646	*cpIs42[Pmex-5::mNeonGreen::PLCδ-PH::tbb-2 3’UTR + unc-119(+)] II; zen-4(or153) IV*	This study
UM655	cpSi20[Pmex-5::TAGRFPT::PH::tbb-2 3’UTR + unc-119 (+)] II; ani-1(mon7[mNeonGreen^3xFlag::ani-1]) unc-119 (ed3)* III; ltIs37 [pAA64; pie-1::mCherry::HIS-58; unc-119(+)] IV	This study
UM657	nmy-2(ne3409ts) I; cpSi20[Pmex-5::TAGRFPT::PH::tbb-2 3’UTR + unc-119 (+)] II; ani-1(mon7[mNeonGreen^3xFlag::ani-1]) unc-119 (ed3)* III	This study
UM735	*xnSi1[Pmex-5::GFP::PH(PLC1delta1)::nos-2 3’UTR] II; estSi71[pAC257;Pmex-5::lifeAct::mKate2::tbb-2 3’UTR; cb-unc-119(+)] IV*	This study
UM761	rrf-3(pk1426) II; ani-1(mon7[mNeonGreen^3xFlag::ani-1]) unc-119 (ed3)* III; ltIs37 [pAA64; pie-1::mCherry::HIS-58; unc-119(+)] IV; ltIs44[pAA173, pie-1p-mCherry::PH(PLC1delta1) + unc-119(+)]	This study

* *unc-119(ed3)* was in the parental strain but may not be present in this strain.
